# Cannabis-induced myocardial infarction in a 27-year-old man: Case report

**DOI:** 10.1016/j.amsu.2022.104054

**Published:** 2022-06-25

**Authors:** Hanane Aissaoui, Soumia Boulouiz, Mohammed El-Azrak, Amine Bouchlarhem, Noha Elouafi, Zakaria Bazid

**Affiliations:** Department of Cardiology, Mohammed I University of Oujda, Mohammed VI University Hospital, Epidemiological Laboratory of Clinical Research and Public Health, Oujda, Morocco

**Keywords:** Acute coronary syndrome, Cannabis, Cardiovascular disease, Case report, Percutaneous intervention

## Abstract

Cannabis smoking has been reported as one of the risk factors for coronary heart disease, which can trigger in rare cases, an acute coronary syndrome (ACS). In this report, we present a case of a 27-year-old man presented with acute myocardial infarction (AMI) following cannabis consumption. The patient developed ST-segment elevation on the anterior and inferior leads. Coronary angiogram demonstrated a significant stenosis of the left anterior descending coronary artery (LAD). A Percutaneous Coronary Intervention (PCI) of the LAD, was realized with the implantation of a new generation-stent with good clinical evolution status.

Healthcare professionals should consider cannabis consumption as a possible etiology of acute myocardial infarction, particularly in young patients with a susceptible social profile (drug-using patients with coronary heredity as a cardiovascular risk factor), and should educate patients regarding this emerging public health issue.

## Highlights

• Cannabis smoking is a known risk factor for coronary heart disease.
• The pathophysiology of myocardial infarction in cannabis users is underreported and multifactorial.
• Acute coronary syndrome in young patients can be challenging due to the wide range of causing differential diagnoses.
• Healthcare professionals should suspect myocardial infarction as a cause of chest pain within young cannabis users.
• The complications are devastating on the quality of life of young patients, and we should raise awareness of his consequences.

## 1 Introduction

Coronary artery disease (CAD) is one of the most common diseases of the elderly population and a major cause of death [1]. Although acute coronary syndrome (ACS) usually occurs in the elderly group, younger people can also be affected [2]. The cardiovascular effects of cannabis have been well documented in the literature. Due to its euphoric effects, it can lead to blood pressure reduction, orthostatic hypotension, and cardiac arrhythmias [3]. It can trigger in rare cases an acute myocardial infarction (MI) and may lead to coronary artery spasm [4].

ACS in young patients can be challenging due to a large range of causing differential diagnoses. The complications of the disease on the quality of life of younger patients are devastating; and there are currently no guidelines or consensus regarding the prevention and treatment of ACS in young patients.

## 2 Case report/case presentation

**Fig. 1:**
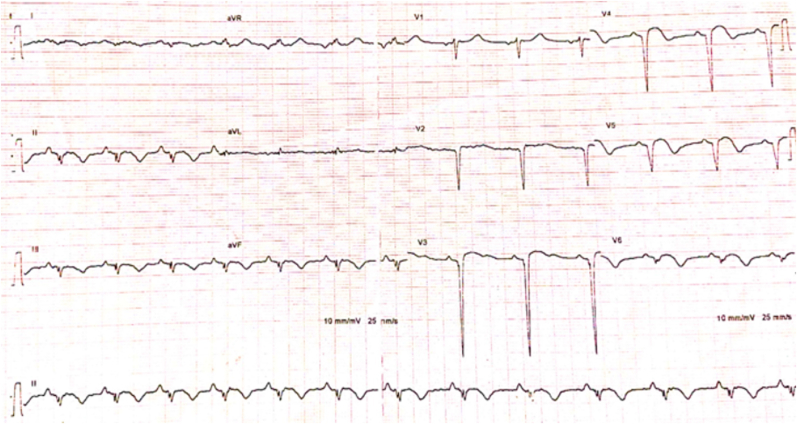
Electrocardiogram showing post-MI on the anterior and inferior leads.

A 27-year-old patient, heavy smoker, long-term cannabis user (for 12 years), and occasional alcohol user. He doesn't have any other risk factors for ischemic heart disease. He denied any other illicit substance abuse, including cocaine. The patient was referred to our department for post-myocardial infarction in the anterior and inferior leads. He reported prolonged chest pain one week before his admission, after cannabis smoking. Physical examination shows a heart rate at 94 bpm, blood pressure at 130/70 mmHg, a temperature of 36.2 C, a respiratory rate of 18 c/m and, and an oxygen saturation of 97%. The cardiac examination showed normal heart auscultation with discrete inferior limb edema. An electrocardiogram (EKG) revealed ST elevation on the anterior and the inferior leads with negative T waves and Q wave necrosis on the same territory (Fig. 1).

Biological analysis revealed an elevated troponin level (249 ng/l), normal CRP range (2.3mg/l) hepatic serology and Covid-19 PCR were negative, with no abnormalities in the lipid metabolic balance, fibrinogen level was normal.

**Fig. 2:**
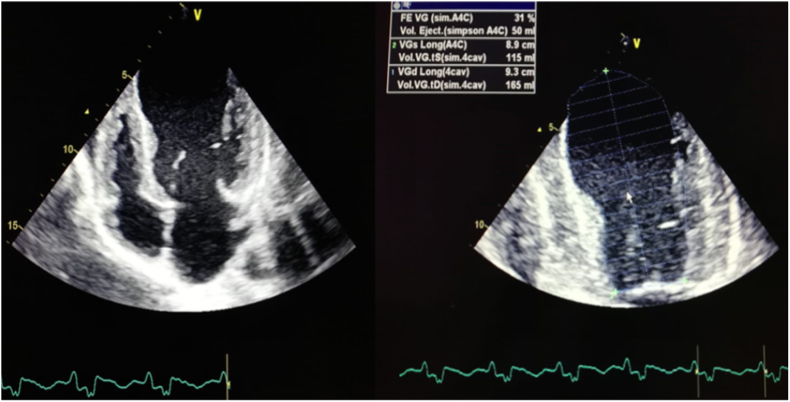
Echocardiography imaging displaying severe systolic dysfunction (EF 31%).

Trans Thoracic Echocardiogram (TTE) showed an aspect of dilated cardiomyopathy (DCM), with a systolic dysfunction (EF at 31%) and a global hyperkinesia (Fig. 2).

**Fig. 3:**
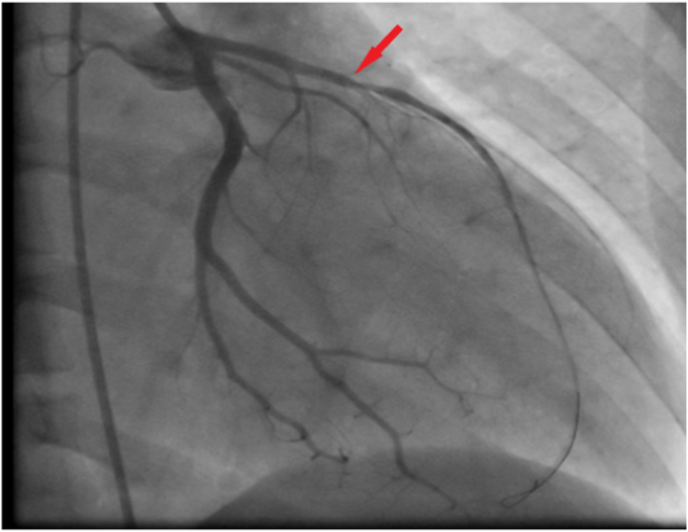
Coronary angiogram showing significant stenosis of the proximal LAD (red arrow). (For interpretation of the references to colour in this figure legend, the reader is referred to the Web version of this article.)

**Fig. 4:**
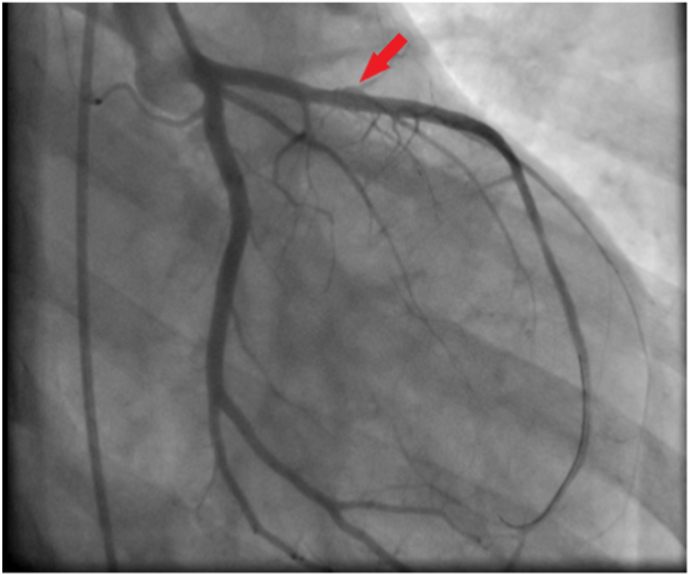
A Percutaneous Coronary Intervention (PCI) of the LAD using a Promus premier stenting (2.75/24mm) (red arrow). (For interpretation of the references to colour in this figure legend, the reader is referred to the Web version of this article.)

Coronary angiography was performed and a significant stenosis in the middle segment of the left anterior descending artery (LAD) was noted (Fig. 3). A Percutaneous Coronary Intervention (PCI) of the LAD was realized with the implantation of a new generation-stent (Fig. 4).

The patient was treated with dual antiplatelet aggregation (Aspirin and Clopidogrel), statins, Beta-blockers, and conversion enzyme inhibitor (CEI), with a good initial clinical evolution.

Follow-up: The patient has not reported any recurrence of chest pain, and his TTE had showed a mild EF amelioration from 31% to 38%, one month after his discharge from the hospital.

## 3 Discussion

Cannabis use increases the risk of cardiovascular events, not only in patients with traditional cardiovascular risk factors but also in the younger population without any risk factors [5]. Herein, we describe the case of a myocardial infarction caused by cannabis use in a young male with no significant past medical history.

51 cases describing cannabis-related MI had been reported in the literature. The first case was described in 2003, since then the number of cases is significantly rising [6]. K. Chetty and reported in the literature review that the mean age of the patients was 31 years. The Majority of them were male (94%). 75% of those patients had no cardiovascular risk factors. 80% presented with chest pain within 6 hours of cannabis use which 71% of them suffered from acute myocardial infarction with ST-elevation [6].

The pathophysiology of SC in cannabis users is not fully understood and it's underreported [7]. However, there is a direct role of endocannabinoids to cause a decreased contractility, and trigger hyperadrenergic state through receptor-mediated and receptor-independent mechanisms [8,9], causing a major release of catecholamines from sympathetic nerve terminals at the myocardium, causing a significant increase in heart rate leading to hypertension [4] and increasing the risk of arrhythmia [10]. At high doses, we observe the reversed effects of marijuana, resulting in bradycardia and hypotension [11].

Also, cannabis smoking causes a net decrease in oxygen supply/demand ratio, which is due in part to an increase in blood carboxyhemoglobin levels [10,11]. It can also cause endothelial dysfunction, platelet activation, and increase ruptured coronary plaque with thrombus formation [11,12]. Coronary vasospasm or slowed coronary flow had been also reported [13,14].

Due to the variety in the underlying mechanisms for cannabis-induced MI, it is likely that the pathogenesis is rather multifactorial and not well understood, requiring more research in the field.

The SCARE guidelines were used in the writing of this paper [15].

## 4 Conclusion

The increasing use of cannabis exposes a major risk for AMI. The physicians should be vigilant in the diagnosis of cannabis-induced MI within young patients, particularly with no cardiovascular risk factors. This case discusses the multiple physiopathological mechanisms by which cannabis can cause myocardial infarction. We hope that it will make the public more aware of the deadly consequences of cannabis use on the cardiovascular system and help to reduce the morbi-mortality rates associated with AMI due to cannabis use.

## Ethical approval

None.

## Sources of funding

None.

## Author contribution

Dr. AISSAOUI, DR. EL-AZRAK, contributed to the literature search, DR. AISSAOUI, DR. BOULOUIZ contributed to the writing, DR. ELOUAFI, DR. BAZID, contributed to the revision, All authors approved the final version of the article.

## Registration of research studies

Name of the registry:

Unique Identifying number or registration ID:

Hyperlink to your specific registration (must be publicly accessible and will be checked):

## Guarantor

AISSAOUI HANANE.

## Consent

The patient has accepted publishing his case and has given his consent.

## Declaration of competing interest

The authors declare no conflicts of interest and no funding resources.
